# From Emergency Care to Community Healing: Developing Culturally Responsive Pathways for Aboriginal and Torres Strait Islander Women with Violence-Related Traumatic Brain Injury

**DOI:** 10.3390/ijerph23040415

**Published:** 2026-03-25

**Authors:** Michelle S. Fitts, Yasmin Johnson, Gail Kingston

**Affiliations:** 1 Menzies School of Health Research, Charles Darwin University, Alice Springs, NT 0810, Australia; 2 Institute for Culture and Society, Western Sydney University, Parramatta, NSW 2150, Australia; johnsonyasmin72@outlook.com; 3 Australian Institute of Tropical Health and Medicine, James Cook University, Townsville, QLD 4814, Australia; 4 Townsville Hospital and Health Service, Townsville, QLD 4814, Australia; gail.kingston@health.qld.gov.au

**Keywords:** women, traumatic brain injury, violence, Australia, Aboriginal and Torres Strait Islander, care systems

## Abstract

**Highlights:**

**Public health relevance—How does this work relate to a public health issue?**

- Physical violence to the head and neck can cause long-term injury and disability among Aboriginal and Torres Strait Islander women, including traumatic brain injury (TBI).
- Although emergency departments (EDs) play a critical role in responding to Aboriginal and Torres Strait Islander women who sustain violence-related TBI, little is known about how hospital and community care can be improved from the perspectives of health professionals working in ED.

**Public health significance—Why is this work of significance to public health?**

- The findings highlight the need for more consistent ED responses, including the implementation of a violence-related TBI protocol and high-quality violence-related TBI training for ED staff.
- A specialised Aboriginal and Torres Strait Islander workforce with expertise in violence and TBI, alongside adequately resourced community-based services, is essential to support women across hospital and community settings.

**Public health implications—What are the key implications or messages for practitioners, policy makers and/or researchers in public health?**

- There is clear need to develop a coordinated pathway from acute care to the community that is tailored to the short- and long-term needs and priorities of Aboriginal and Torres Strait Islander women following violence-related TBI.
- Policymakers and practitioners may use these findings to strengthen identification, response, and treatment practices across hospital and community settings.

**Abstract:**

Emergency departments (EDs) are critical points of contact for treating and responding to the needs of Aboriginal and Torres Strait Islander women who have experienced traumatic brain injury (TBI) caused by violence. This study aimed to explore how care, support, and treatment can be improved for Aboriginal and Torres Strait Islander women who have experienced TBI caused by violence by drawing on the perspectives of ED staff in a regional hospital in Queensland (Australia). Using purposeful and snowballing sampling, 24 health professionals (including Indigenous hospital liaison officers and Aboriginal health workers and nursing, medical, and allied health staff) were recruited to participate in the study. Using reflexive thematic analysis, four key recommendations were identified: (1) development of a formalised pathway for head injury from family violence; (2) providing Aboriginal and Torres Strait Islander women with a timely acute-care-to-community pathway; (3) resourcing hospital- and community-based services for all Aboriginal and Torres Strait Islander women and their children; and (4) strengthening responses from health professionals to violence and head injury. The findings inform opportunities to strengthen ED and system-level responses to improve care and support for Aboriginal and Torres Strait Islander women who have experienced this injury.

## 1. Introduction

Traumatic brain injury (TBI), a leading cause of disability, can have devasting, life-long effects on the day-to-day lives of people who sustain the injury, their families, and wider communities [1]. TBI is defined as damage to, or alteration of, brain function due to a blow to the head or force applied to it [2]. In the context of violence, TBI may also result from deprivation of the supply of oxygen to the brain caused by non-fatal strangulation (e.g., hypoxia or ischemia). TBI can be classified as mild, moderate, or severe, based on clinical indicators such as level of consciousness, duration of memory loss, and neuroimaging results. Although the experience of TBI is unique to each person, common effects include changes in thinking, memory, emotions, behaviour, and physical functioning. Symptoms may appear immediately or emerge over time.

In Australia, Aboriginal and Torres Strait Islander peoples experience a disproportionately high incidence and prevalence of TBI [3,4,5,6], contributing substantially to the burden of disability in this population [7]. In north Queensland, the rate of TBI among Aboriginal and Torres Strait Islander peoples is approximately twice that of non-Indigenous populations [3]. While TBI prevalence data is sparse in Australia [8,9], available evidence indicates that assault is a leading cause of TBI among Aboriginal and Torres Strait Islander peoples, particularly women [4,5]. An Australian study examining data from Queensland, Western Australia, South Australia, and the Northern Territory (1999–2005) found that the proportion of assault-related head injuries among Aboriginal and Torres Strait Islander women was 69 times higher compared to that among other women [5]. Violence-related head injuries are often frequent and repetitive, with little time for recovery between incidents [10,11]. Addressing TBI in the context of violence is therefore a critical concern reported by Aboriginal and Torres Strait Islander communities, particularly Elders [12].

Healthcare services, in emergency departments (EDs) in particular, are uniquely positioned to support Aboriginal and Torres Strait Islander women who present with TBI resulting from violence. ED processes and protocols are designed to rapidly identify and manage injuries that pose a risk of permanent disability or death. Once a serious injury is ruled out, EDs may initiate a multidisciplinary response to aid individuals in accessing additional services. Many ED health professionals view these presentations as opportunities to address immediate injuries, provide safety, connect Aboriginal and Torres Strait Islander women with support services, and share information on family violence and TBI [13,14]. Despite this, there remains limited evidence of how ED service systems can best deliver treatment and care to Aboriginal and Torres Strait Islander women presenting with a TBI caused by family violence [14,15]. Aboriginal and Torres Strait Islander peoples with TBIs often experience difficulties within ED environments that are not responsive to cognitive, sensory or emotional symptoms associated with brain injury. Challenges in navigating fragmented service systems further contribute to disengagement from care [10,15].

To address these gaps, specialised roles such as Aboriginal brain injury coordinators and nurse navigators have been recommended and trialled [16,17,18]. Nurse navigator models offer continuity of care by providing a single, consistent point of contact across the patient journey, supporting authentic, non-judgmental, and culturally responsive care [18]. For Aboriginal and Torres Strait Islander women, whose care and support needs are often complex, such models are particularly critical.

Research on TBI has historically demonstrated a significant gender disparity, with most studies focusing on the experiences of men [19]. Alongside increasing policy attention to the prevention of and response to family violence, there is a growing need to understand the experiences of women living with TBI resulting from violence [10,20,21,22,23,24]. Evidence suggests that women experience poorer outcomes than men following TBI [25]. Over the past decade, research in Australia and worldwide has increasingly documented women’s lived experiences of navigating service systems and adapting to life after TBI, with the aim of driving effective and meaningful change [10,26,27]. Without support specifically designed for women—and responsive to gendered roles and caregiving responsibilities—women are at an increased risk of long-term adverse outcomes following TBI.

### 1.1. Study Aim

This study formed part of a larger, multi-jurisdictional, three-year project that aimed to understand the needs and priorities of Aboriginal and Torres Strait Islander women who have experienced TBI caused by violence [28]. The specific aim of this study was to explore the experiences and perspectives of health professionals working in a regional ED regarding how treatment of and care for Aboriginal and Torres Strait Islander women presenting to the hospital with head injuries caused by family violence can be improved. During the initial phase of the project, guidance and advice were provided by Elders, community members, senior leadership from the hospital, and community-based services [29]. Based on this advice, it was recommended that recruitment focus solely on ED healthcare professionals, as most Aboriginal and Torres Strait Islander women with violence-related TBI receive all of their care and treatment within the ED setting. These recommendations, together with lived-experience expertise from Aboriginal and Torres Strait Islander women [10], can inform the development of rehabilitation approaches that are timely, culturally responsive and fully accessible.

## 2. Methods

### 2.1. Study Setting and Ethical Considerations

One regional hospital in north Queensland, Australia, participated in this study. The hospital is responsible for the delivery of healthcare services, education and research. To maintain the anonymity of participants, the location of the hospital is not disclosed in this article. Ethical approval for this study was received from the local Hospital and Health Service Human Research Ethics Committee (HREC/QTHS/85271), in addition to site-specific approval from the participating hospital. The research was guided by the National Health and Medical Research Council’s values and ethics for completing research alongside Aboriginal and Torres Strait Islander communities [30].

### 2.2. Study Design

This study uses a qualitative descriptive design to draw out the key health, cultural, and social determinants of appropriate care as determined by ED professionals for Aboriginal and Torres Strait Islander women [31]. This approach is useful when there is a lack of research on a topic area and offers a ground-up approach to collecting new data to strengthen findings and implications.

### 2.3. Participants and Sampling

A wide range of professionals were recruited to acquire a deep and detailed understanding of the care women receive. Staff across key professions (e.g., medicine, nursing, social work, occupational therapy, and physiotherapy) and specialised healthcare workforce staff, such as Indigenous Hospital Liaison Officers (IHLOs) and Aboriginal Health Workers (AHWs), were invited to participate in a face-to-face interview or focus-group discussion. Individual and dyadic interviews and focus-group discussions were facilitated by a non-Indigenous woman (author: MF) and Aboriginal woman (author: YJ) [32]. Data collection was completed between September and November 2022. Eligibility criteria required individuals to be aged 18+. Frontline staff with both short- and longer-term experience working at the hospital and ED were included to capture a broad range of experiences, with no restrictions on the length of employment at the regional hospital or prior experience working with women affected by violence-related TBI.

In total, 24 health professionals participated in the study. Thirteen staff participated in an individual interview. A further four staff members participated in dyadic interviews. Seven staff members participated in focus groups. The overall sample included nine IHLOs and AHWs, eight medical staff (including doctors and nurses), and seven allied health professionals (including social workers, occupational therapists, and physiotherapists). Eleven health professionals who participated in the study identified as being of Aboriginal and/or Torres Strait Islander background. Eighteen of the health professionals who participated in the study were women, and six were men. Health professionals had been working in the hospital for an average of 5 years (range: 1 to 20 years).

At the preference of the health professionals, interviews and focus-group discussions took place at a location within the hospital (e.g., private room at the library). Except for one individual interview and one paired interview, all interviews were recorded using an audio recorder and then transcribed verbatim by a professional transcription company. For the one individual interview and one paired interview, written notes were recorded by the research team members and typed up into individual Word documents upon completion of the interviews.

### 2.4. Positionality Statement

As researcher positioning is an essential component to conducting reflexive, ethical and high-quality research, the authors embedded within the research process describe their worldview and positionality. The first author is a white settler, who was raised on Bunurong Country, and now lives and works on Arrernte Country. Through decolonised research practices, she is committed to elevating the voices of Aboriginal and Torres Strait Islander women who have experienced TBI from violence, as well as those of their families and communities. This work aims to strengthen and inform the development of health, justice and child protection systems so they are more responsive to TBI. The second co-author is a proud Wulgurukaba, Ngaro and Goreng Goreng (saltwater) Aboriginal woman who has 20 years of experience working within the social services sector in early family crisis intervention work and, more prominently, in the space of family violence advocacy. She applies a trauma-informed, person/family-centred approach and operates through the lens of embedding healing practices and cultural safety within contextualised frameworks when working within communities. The third author is a non-Indigenous woman who was raised on the country of the Eora nations. For over 30 years, she has worked in tertiary healthcare as an occupational therapist as well as a lecturer in both Occupational Therapy and Public Health. With a passion for developing acute care and rehabilitation models of care for Aboriginal and Torres Strait Islander peoples who sustain injuries in regional and remote areas, she has been an advisor on several large research projects across northern Australia, including on TBI and telehealth.

### 2.5. Data Collection

Purposeful and snowballing sampling techniques were used to recruit health professionals for this study. First, copies of the participant information sheet and consent form were emailed to team leaders, including a summary of the project and the contact details of the lead researcher. Staff interested in participating in an interview or focus-group session contacted the lead researcher either via telephone or email. Once contact had been made, the researcher liaised with each potential participant to find a suitable time to meet in person to discuss the information disseminated about the project (hard copies of the participant information sheet and consent form) and, if consent was given, to conduct an interview or focus-group session. Recruitment also involved attendance at staff meetings and clinical education sessions (face-to-face and online) to present information on the project and advise avenues of staff participation if interested. Snowball sampling, in which health professionals recommended other relevant staff to participate, was also used [33]. Some health professionals were recommended to the research team and therefore contacted directly. As described in the study protocol, a flexible interview guide was used to focus the conversation on key questions pertaining to factors that enhanced or challenged healthcare and interactions women had at the hospital as well as policies and practices that could be implemented to improve the healthcare women receive in the hospital and after completion of treatment [34]. Example questions included the following: Can you tell me about Aboriginal and Torres Strait Islander women who present to hospital with a head injury because of family violence?; What is working well in terms of your service delivery? and What are the major challenges to your service delivery?. Written consent was given by each participant. With the permission of health professionals, handwritten notes were also taken by the researchers to enable recall of particularly pertinent sections of the discussion for later consideration [35]. Although health professionals were asked to share their perspectives and experiences of providing care and treatment to Aboriginal and Torres Strait Islander women with TBI caused by family violence, several health professionals also reflected on their own journeys of family violence. All interviews, except one individual interview and one dyadic interview, were recorded using a digital audio recorder. All participant data were kept confidential, with only those members of the research team who were not directly affiliated with the hospital having access to transcripts and recordings.

### 2.6. Data Analysis

The audio recordings of the interviews and focus groups were transcribed by a professional transcriber into separate Word documents, assigned a number, and checked for accuracy against the original recordings. Written notes from the interviews and focus groups that were not audio-recorded were typed and stored in separate Word documents and assigned a number. Transcripts were stored and managed using NVivo V.12. Transcripts were coded using reflexive thematic analysis, as described by Braun and Clarke [36]. This approach enabled the research team to utilise an open iterative approach to coding. Codes were derived directly from the experiences of health professionals in the hospital rather through than a fixed framework at the start of the process [37]. The first phase of analysis involved reading and re-reading the transcripts and listening to the audio recordings to increase familiarity with the data. Once familiar, the coders (authors MF and YJ) generated an initial list of ideas and identified early concepts for coding. Initial ideas were discussed between the authors, and when an agreement was reached, codes were organised into groups and initial themes were developed. The next phase involved reviewing the data extracts coded under each theme and deciding whether or not they fit within the theme and if they formed a sound and logical pattern. Independently, each coder completed this process before reaching a consensus and amending NVivo. This process was repeated; the coders re-read the transcripts and coded any additional material with the new codes. In the next phase, each theme was renamed, defined, and summarised. Acknowledging researcher impact on data interpretation [38], the authors presented the initial themes to a selection of frontline and senior management staff in meetings and presentations to ensure the accuracy of the quotes and naming of the themes. Discussions during these meetings and presentations led to further refinement of the coding. All extracts were checked for accuracy. The final phase involved writing up the findings to reflect the interpretation of the data. The data presented in this study are not publicly available due to ethical restrictions and the need to protect confidentiality.

## 3. Results

Four themes describing health professionals’ perceptions of how existing responses to Aboriginal and Torres Strait Islander women who present to the ED with a TBI caused by family violence could be improved emerged from the data analysis: development of a formalised pathway for head injury from family violence; providing Aboriginal and Torres Strait Islander with a timely acute-care-to-community pathway; resourcing hospital- and community-based services for all Aboriginal and Torres Strait Islander women and their children; and strengthening responses from health professionals to violence and head injury. These themes, which are illustrated by quotes from health professionals, are outlined below.

### 3.1. Development of a Formalised Pathway for Head Injury from Family Violence

Health professionals suggested developing a formalised care pathway, with the expectation that it would lead to a standardised response issued by the hospital system for all Aboriginal and Torres Strait Islander women presenting to the ED with a TBI caused by family violence. This was perceived as a way to address inconsistent responses that may arise from the variation in clinicians’ skills and knowledge, personal beliefs about family violence, and awareness of the multidisciplinary healthcare services available within the hospital. Allied health staff also noted that a standardised pathway of care could increase the number of referrals to social work and occupational therapy: *“If we had clearer guidelines around our referral process for those patients, we would pick up those referrals a lot easier”* (participant 16, Allied Health, Interview).

When discussing the pathway for TBI caused by family violence, healthcare professionals from all the participant groups drew on existing ED pathways, including those for sexual assault and other conditions, including stroke and alcohol withdrawal. Elements of the sexual-assault pathway were considered particularly relevant, including clinical prompts and clear role delineation for health professionals to ensure that appropriate treatment and referral pathways are consistently identified and actioned: *“This is what you do, this is who you ring, this is what you say or don’t say, leave it at that. Tick the boxes, so that the right people are involved at the right time”* (participant 21, Medical and Nursing, Interview). As another participant extrapolated:

*“Like, the sexual-assault pathway in itself is four pages. But yeah, the creation of that would be fantastic, just to try and actually have something that people could be like, Oh, well, this person’s been assaulted, how do we then make sure that this person is going to be safe going home?”*

(participant 19, Medical and Nursing, Interview)

Advocating for the need for a formalised pathway for Aboriginal and Torres Strait Islander women presenting with injuries caused by family violence, one participant stated, *“These women don’t get any additional support. The system is just not willing to accommodate these women”* (participant 1, Medical and Nursing, Interview). Participants identified several elements of the existing sexual-assault pathway that were considered critical for inclusion in a TBI and family-violence pathway. These included access to a worker with specialised training, 24 h operation of the pathway, fast-tracking of Aboriginal and Torres Strait Islander women upon presentation to the ED, and the provision of a safe and trusted space. Other key components included victim/survivor-centred responses support for patient choice (for example, considering the gender of the medical examiner) and appropriate follow-up care post-discharge from the hospital to minimise retraumatisation. The following quote illustrates how responses to injury within the formalised sexual-assault pathway differ from those provided in the family-violence context:

*“Immediately, there’s a level of confidentiality straight away. Immediately, staff become more conscious about who’s going into that room—male [or] female clinicians. There’s a sexual assault nurse who’s called to support that person. The social worker may be called to support that person. Then they have police and everything contact the department and that person is given well-rounded support. The medical team have a very defined role in that instance, which is to make sure that she’s got no overt injuries; to offer her contraception; to offer her treatment. She has a very clearly defined pathway with supports that are escalated straight away. […] Domestic violence is treated the complete opposite. There’s no additional confidentiality. There’s no additional supports. There’s no additional escalation. It’s actually just treated as if she has come in with abdominal pain. The differences between the two are very stark.”*

(participant 14, Medical and Nursing, Interview)

Although standardisation of treatment and care was recommended, health professionals suggested that the pathway would need to have in-built flexibility to support Aboriginal and Torres Strait Islander women and that the treating team would contribute to the tailoring of an individualised care plan.

### 3.2. Providing Aboriginal and Torres Strait Islander Women with a Timely Acute-Care-to-Community Pathway

Providing integrated care for violence-related TBI through the development of a care-to-community pathway was a frequently mentioned recommendation from health professionals. Most Aboriginal and Torres Strait Islander women who present to the ED with head injuries due to violence received all their treatment and care within the ED setting. Based on the accounts from health professional, this places significant pressure on frontline staff to not only assess and treat Aboriginal and Torres Strait Islander women for their injuries but also supply information about brain health, head injuries, and how to manage ongoing symptoms as well as assist them with their accommodation, financial, and safety needs. Some hospital social workers expressed concerns about how much information Aboriginal and Torres Strait Islander women could meaningfully retain in relation to their safety plans and follow up with referrals after discharge from the hospital. These concerns related to Aboriginal and Torres Strait Islander women managing common post-TBI symptoms, such as changes in concentration and memory.

Different models for post-discharge support for Aboriginal and Torres Strait Islander women who had experienced violence-related TBI were discussed. One suggestion was to build on an existing coordinated care model implemented in the hospital—specifically the appointment of nurse navigators, who provide outreach support to people with a history of multiple ED presentations and unplanned admissions. Some health professionals further suggested creating a dedicated nurse navigator role with specialised training in family violence and TBI:

*“We do have nurse navigators, they’re fantastic. So whether there—it could be kind of linked into the nurse navigation service as an additional thing—but whether there could be creation of an additional nurse navigator role to follow up with these patients long term and see how they’re going.”*

(participants 16–18, Medical and Nursing, Focus Group)

Other ideas on ways to strengthen the acute-care-to-community pathway included co-locating community-based organisations within the hospital, such as local Aboriginal Community-Controlled Health Services and family-violence service providers. As the following quote suggests, Aboriginal and Torres Strait Islander women could meet a case worker while in the hospital and then be followed up by the same case worker in the community:

*“If you had NGOs (non-government organisations) based in the hospital, where we can connect them [women] through to them, who will then connect them back and follow that connection in the community, I mean, that’s the easiest way that we can provide better support around our mob coming in around this issue.”*

(participants 4–6, IHLO and AHW, Focus Group)

### 3.3. Resourcing Hospital- and Community-Based Services for All Aboriginal and Torres Strait Islander Women and Their Children

#### 3.3.1. Investment in Hospital Services

All health professional participant groups recommended greater financial investments across hospital service systems to improve service accessibility. Within the hospital setting, health professionals emphasised the need for cultural and social support teams (e.g., social workers and IHLOs) to be available during peak presentation periods such as nights and weekends. At the time of data collection, hospital-based social and cultural resources were limited during these high-demand times for TBI presentations in the ED, with after-hours support often restricted to on-call arrangements and coverage across the entire hospital:

*“I think it needs to change to an ED where if they are in triage, there’s a certain amount of time that they be seen by one of our mob. And possibly have us working 24/7 in the hospital, like in ED at least. There’s no night shift, nothing. [But] the majority of our people present after hours. Especially with head injuries.”*

(participant 1, Medical and Nursing, Interview)

As a result, there was often minimal engagement with the hospital-based services available to support Aboriginal and Torres Strait Islander women who had experienced TBI as a result of violence.

#### 3.3.2. Spaces for Children in the Hospital

Health professionals also felt that hospital space and service redesign could better support the everyday care responsibilities of Aboriginal and Torres Strait Islander women and their children during hospital stays as well as improve transitions from hospital to community-based care after discharge. In discussing system-level improvements, the health professionals reflected on the experiences of Aboriginal and Torres Strait Islander mothers and the long-term intergenerational impacts that family violence and TBI can have on both mothers and their children. To support engagement with existing hospital services, health professionals recommended providing accommodation connected to the hospital as well as on-site childcare facilities. As one participant stated, *“The dream would be building houses, having day cares”* (participants 4–6, IHLO and AHW, focus group). Another participant made the following suggestion:

*“Yeah, a phone and just our own little shelter that we could have something that was manned from whatever time, because after hours, everything stops. So, as soon as it gets to 5:00 in the afternoon, absolutely there’s nothing.”*

(participants 2 and 3, Allied Health, Dyadic Interview)

At the time of data collection, the regional hospital had access to an existing outsourced childcare facility. According to the health professionals’ accounts, this service was usually at full capacity. Childcare and child-friendly spaces were described by health professionals as a crucial healthcare requirement for Aboriginal and Torres Strait Islander women experiencing family violence to support them to participate fully in decision making regarding their healthcare journey:

*“Quite often those women won’t want to stay because they’ve got conflicting responsibilities with children. Many children have been put with a relative who they wouldn’t ordinarily leave their children with. The children are still in the house with the perpetrator of the violence. So quite often, these women leave and go back to that situation.”*

(participant 20, Medical and Nursing, Dyadic Interview)

#### 3.3.3. Accommodation and Crisis Housing

The availability of accessible and appropriate short-term accommodation for Aboriginal and Torres Strait Islander women and their children outside of the hospital was also noted as being critical to improving healthcare and reducing the risk of further TBI related to family violence. Crisis and short-term accommodation in the region were mentioned as frequently at capacity or inaccessible after hours. As a result, and in the absence of alternative options, Aboriginal and Torres Strait Islander women were sometimes discharged from the hospital to homelessness. As one participant explained, *“If they’ve stayed in hospital, they’re discharged to homelessness, which can be just a bus dropping them off with their belongings. People get dropped in hospital gowns”* (participants 16–18, Medical and Nursing, Focus Group). Health professionals also recommended crisis accommodation options that allowed boys of all ages to access safe housing. At present, many women’s shelters in the regional centre do not accept referrals for women with boys aged above 12+ to stay in crisis accommodation. Consequently, women often have to make alternative arrangements for their children (e.g., having them stay with family), and such arrangements are typically short-term and unstable.

#### 3.3.4. Community-Based Service Providers That Are Accessible After Hours

After-hours accessibility challenges also extended to the community-based services, which were often not funded to operate outside normal business hours. As a result, health professionals had limited options for making direct referrals for Aboriginal and Torres Strait Islander women who presented to the ED outside of business hours. In some cases, Aboriginal and Torres Strait Islander women left the ED with little or no follow-up referrals or immediate support in place:

*“Services within the community are very similar. So, when is DV happening? After hours. So, there’s less support available because there are no services running at that hour. It gives people time to think about what’s going on, I need to get home anyway, so there is less ability to help when they come in through after-hours timeframes because there are no services.”*

(participants 4–6, IHLO and AHW, Focus Group)

#### 3.3.5. Services for the LGBTQIA+ Community

Health professionals also highlighted the need for service provision to be appropriate and safe for the needs of all people who suffer head injuries related to family violence, including those from the LGBTIQA+ community. As one participant noted, “*Having a deliberate service in the community [for queer women]. We don’t seem to really have one, or one that I’m aware [of]”* (participant 19, Medical and Nursing, Interview).

### 3.4. Strengthening Responses from Health Professionals to Violence and Head Injury 

#### 3.4.1. Addressing Racism and Bias in Health System Responses 

Health professionals highlighted the need for hospital systems to be more responsive to the intersecting influences of gender and racial background when responding to Aboriginal and Torres Strait Islander women presenting to the ED with TBI related to violence. Health professionals emphasised the importance of providing comprehensive, high-quality education and training to strengthen trauma-informed care, improve understanding of family violence, and address racism within healthcare settings. Health professionals reported that prejudiced assumptions held by some staff contributed to differences in treatment and care, which in turn shaped negative healthcare experiences for Aboriginal and Torres Strait Islander women. As one health professional shared about her own experience of seeking care at the hospital for violence-related injuries, *I was in a domestic violence relationship when I came here because I needed, my nose was broken, and I sat there and the, I think he was a [name of role], and he said “oh you guys are used to this aren’t youse.” I said no I’ve just been through domestic violence and had my nose broken, and I said and “I work here and I can, and I can see very clearly now why my patients don’t want to come.” Like that’s coming from a person that works here. But little did he know that, you know he didn’t know at all, but that’s the sort of, that’s what’s attached to us, as Indigenous people.*

Some health professionals also noted that the injuries and pain experienced by Aboriginal and Torres Strait Islander women can sometimes be dismissed by hospital staff. Such experiences can influence Aboriginal and Torres Strait Islander women’s decisions about whether to seek healthcare in the future. Health professionals suggested that staff training needed to challenge misconceptions and stereotypes surrounding family violence. One example given by health professionals was the minimisation of victim-survivor experiences within LGBTIQA+ relationships, *“Almost like it’s a discountment [sic], if it’s like, if they’re queer. Because it’s like, Oh well, it’s female on female violence. And it’s like, Well, no guys, it’s still domestic violence”* (participant 19, Medical and Nursing, Interview).

#### 3.4.2. Building Health Professional Capacity to Recognise and Respond to Violence-Relataed Head Injury

While some training opportunities currently exist, health professionals reported that these were often limited—for example, brief online courses—and are insufficient to create meaningful change within healthcare settings. Participants therefore identified a need for more in-depth, practice-based training and ongoing support for staff. Some health professionals emphasised that practical skills training is essential for building staff confidence and capability, enabling them to identify signs and signs of both TBI and family violence, establish trust and rapport with Aboriginal and Torres Strait Islander women patients, ask sensitive questions safely, and respond appropriately. As one participant stated,

*“I always want to know what questions should I be asking and when and what are some maybe more subtle signs that someone may be experiencing DV. Maybe if we had a bit more training, I can go in there with a thought process, “Okay, this person’s come in here with x injury. So, these are questions I should be asking.” But as a [health professional], I find it hard to know what questions to ask.”*

(participant 11, Allied Health, Interview)

Allied health professionals also indicated a need to include comprehensive information in training about the services they provide—such as occupational therapy and physiotherapy—for Aboriginal and Torres Strait Islander women who have experienced a head injury, along with guidance on appropriate assessment tools for assessing head injury. Health professionals recommended that hospitals should allocate dedicated time for staff to complete comprehensive training, as a lack of time was a barrier for completing current programs and courses. Targeted training was also seen as a way to improve communication between medical staff and social workers. As one participant said, *“Doctors aren’t always great at referring head injuries to us and that’s about education, awareness, and our role, understanding what role we can have to play with those [women]”* (participant 15, Allied Health, Interview). Another agreed: *“A lot of doctors don’t understand what we do. A lot of doctors don’t understand what is social work. Then because of that, they’re less likely to refer to us”* (participant 12, Allied Health, Interview).

## 4. Discussion

This study builds upon previous research as part of a larger, multi-jurisdictional, three-year project that aimed to understand the needs and priorities of Aboriginal and Torres Strait Islander women who have experienced TBI from family violence [28]. By exploring the experiences and views of health professionals who work in a regional ED, we have developed recommendations regarding the treatment of and care for Aboriginal and Torres Strait Islander women who present to the hospital with head injuries caused by family violence. The findings provide important insights into the resources critical for responding to Aboriginal and Torres Strait Islander women who present to the ED with potential TBI from family violence. Our study identified four overarching areas requiring investment and development to ensure there is a standardised and effective response to Aboriginal and Torres Strait Islander women with this injury while also enhancing the care already offered in the ED. The suggestions offered indicate the Australian healthcare system must move beyond the biomedical response to one that considers the gendered and racialised context of family violence [39]. When a TBI is understood within the broader context of disability and family violence, existing ED responses are too narrow to meet the needs of all Aboriginal and Torres Strait Islander women, potentially undermining their effectiveness. Challenging current practices by thinking about how TBI is defined, understood, and addressed in the context of Aboriginal and Torres Strait Islander women could lead to markedly different responses, including alternative care settings, more in-depth and meaningful conversations, personalised support, and ultimately improved outcomes.

A coordinated care pathway could improve the consistency of responses for Aboriginal and Torres Strait Islander women who present to the ED, especially by establishing protocols specific to Aboriginal and Torres Strait Islander women who have experienced TBI from family violence, including providing access to private space. As the participants in this study highlighted, integrated and coordinated care pathways already exist for other conditions, including stroke, as well as other violence-related injuries, including sexual assault [40,41,42]. Further research is recommended to co-design and trial a coordinated care pathway for violence-related TBIs in regional and remote EDs, informed by the lived experiences of Aboriginal and Torres Strait Islander women who have experienced TBI due to violence. Racialisation and discriminatory practices continue to occur in healthcare settings with respect to marginalised populations with TBI [43]. Therefore, it is essential to centre the voices of Aboriginal and Torres Strait Islander women and develop unique approaches to establishing coordinated care pathways, services, and community integration, particularly regarding innovative access points for treatment.

Following the acute-care phase, a supported care journey may help Aboriginal and Torres Strait Islander women navigate community-based service systems that are often designed to address needs related to TBIs or family violence, but not both [14]. Drawing on earlier studies, examples of such support include brain- and spinal-injury clinic pathways, Aboriginal brain injury coordinators, and nurse navigators [16,17,18]. Health professionals included in this study also suggested co-locating key community-based services within the hospital to strengthen connections between hospital referrals and ongoing community support. Future research is needed to determine the skills and qualities required for staff in these roles. A variety of factors can influence when and how Aboriginal and Torres Strait Islander women access hospital care. Researchers have stressed that Aboriginal and Torres Strait Islander women who do not access hospital care should still be able to access intensive support through referrals to trained caseworkers [10].

If a standardised hospital pathway, combined with a specialised worker to support Aboriginal and Torres Strait Islander women after the acute phase, is to be implemented, longstanding systemic and structural inequalities must be addressed. Health professionals’ accounts highlighted the need for greater investment in hospital- and community-based services after hours as well as in childcare and housing. This includes both crisis accommodation and longer-term options that could provide Aboriginal and Torres Strait Islander women with greater stability and independence.

Although alternative accommodation is critical for women leaving relationships characterised by violence, it is often unavailable due to high demand and insufficient resources [44,45]. Increased access to both crisis and longer-term housing options within the community would allow Aboriginal and Torres Strait Islander women and families to focus on healing and rebuilding stability. Evidence from Canada suggests that long-term or ‘second stage’ options following crisis housing can foster women’s independence and support effective transitions towards living well after violence [46].

Consistent with the previous literature, health professionals reported Aboriginal and Torres Strait Islander women without immediate accommodation options often return to the location where their injuries occurred. In these circumstances, the risk of ongoing violence is high, increasing the likelihood of further injury, disability, and death. As identified in this study and elsewhere [45], Aboriginal and Torres Strait Islander women also face barriers to accessing crisis accommodation due to eligibility guidelines related to boys aged over 12 years. Accessible and culturally appropriate services are therefore critical to supporting Aboriginal and Torres Strait Islander women’s recovery following TBI from violence.

Capacity building is frequently identified in the literature as essential for developing knowledge and skills required for responding effectively to family violence as well as transforming attitudes and values to support gender and racial equity in service provision. Existing research highlights the persistence of stereotyping and bias in responses to family violence—including victim-blaming narratives that position Aboriginal and Torres Strait Islander women as responsible for their experiences of violence and the question ‘why does she not leave?’ [47]. Levels of family-violence training among ED staff can vary widely [48]. However, capacity building cannot be limited to a single training session, as brief educational interventions may improve knowledge but rarely lead to sustained changes in behaviour [49]. Ongoing support and reinforcement are therefore required in order to develop and maintain healthcare professionals’ competencies and should be embedded within continuing professional development programs.

### 4.1. Limitations

This study involved a single regional hospital with 24 participants at one timepoint, with no practice observation data. As a result, the findings may not reflect experiences in other EDs in Australia or internationally. While the perceptions and recommendations generated in this paper come from the perspectives of staff working in the ED, it is important to acknowledge that some healthcare professionals spoke not only about their experiences as healthcare professionals but their own lived experiences of violence and TBI from violence. Finally, data collection was completed during the COVID-19 pandemic, and this may have affected staff availability for participation in the study.

## 5. Conclusions

There is a growing body of research in Australia and worldwide demonstrating the need to develop alternative approaches to improve treatment and care for women who present to hospital with TBI from violence. The findings of this study offer initial guidance for the development of an approach to support Aboriginal and Torres Strait Islander women in recognising and engaging with the inherent connections between individual circumstances and intersecting social identities, particularly within the context of regional Australia. These recommendations may help ensure that Aboriginal and Torres Strait Islander women receive a consistent and holistic response that addresses their health, cultural, and social needs. Both TBI and family violence have long-lasting impacts on health and lead to multiple forms of disability. Aboriginal and Torres Strait Islander women experience some of the highest rates of TBI related to family violence in Australia, underscoring the need for immediate, well-resourced, and coordinated responses.

## Data Availability

The data presented in this study are not publicly available due to ethical restrictions and the need to protect participant confidentiality.
